# Filler in the Tear Trough and Lid-Cheek Junction: A Cadaveric Evaluation of Filler Type and Manipulation Technique

**DOI:** 10.1093/asjof/ojad027.001

**Published:** 2023-04-14

**Authors:** Yoshiko Toyoda, Stephanie Honig, Alexander Wilson, Robyn Broach, Ivona Percec

**Affiliations:** University of Pennsylvania, Philadelphia, PA; University of Pennsylvania, Philadelphia, PA; University of Pennsylvania, Philadelphia, PA; University of Pennsylvania, Philadelphia, PA; University of Pennsylvania, Philadelphia, PA

## Abstract

**Goals/Purpose:**

The tear trough and lid-cheek junction demonstrate among the earliest and most noticeable signs of aging as a result of thin periorbital skin and loss of volume causing shadowing and darkness of the infraorbital region. Thus, rejuvenation of this area is commonly requested by patients. Soft tissue filler injections are the second most common non-surgical cosmetic procedure performed in the U.S, with over 2.6 million procedures reported in the 2020 Plastic Surgery Statistics Report. As such, there is a high demand for filler injection in the infraorbital area. However, there is currently only one FDA-approved filler for the tear trough, Juvéderm Volbella XC, although many practitioners also use Restylane-L. Interestingly, these two products differ significantly in their rheology (G’, cohesivity, and flexibility), and the Juvéderm Vycross is smooth gel while the Restylane NASHA is particulate. Despite the high demand of filler in the infraorbital area, there remains debate on injection practices and precise anatomical placement leading to several prior cadaveric examinations of the region. We herein aimed to contribute to the anatomic understanding of infraorbital filler injection via a cadaveric study using Juvéderm and Restylane products with different constitutions and manipulation techniques.

**Methods/Technique:**

Institutional approval for cadaveric study was obtained. Six cadaveric cephalus specimens were used for filler injection and dissection. All fillers were dyed with food coloring. The Juvéderm products Volbella XC (tear trough, red) and Voluma XC (malar, blue) were used together in hemi-faces (Juvéderm pair), and Restylane products Restylane-L (tear trough, purple) and Restylane-LYFT (malar, green) were used together in separate sets of hemi-faces (Restylane pair). All filler injections were performed by the senior author as follows: 0.5 cc of filler was injected into the malar region in the deep fat compartment above the maxilla both medial and lateral to the infraorbital foramen. In the tear trough region, the orbit was protected with the nondominant hand and 0.3 cc of filler injected just superficial to the infraorbital rim. One hemi-face was injected with the original constitution of filler product and the other was injected with filler blended with 0.2 cc of saline. In two cadavers, after injection the hemifaces were massaged and compared to those which remained unmanipulated. All hemi-faces were then dissected from medial to lateral in the subcutaneous plane and between the superficial and deep facial fat compartments by a single author. All photographs and videography were taken by a single author. All hemi-faces were subjectively analyzed by all authors.

**Results/Complications:**

All cadavers were in excellent condition, and the subcutaneous tissue, superficial fat, and deep fat compartments were dissected in clear planes. Volbella was found on the periosteum of the infraorbital rim and along the orbicularis retaining ligament (ORL) and crossed into the sub-orbicularis oculi fat (SOOF) and the subcutaneous fat while the Restylane-L did not leech into the fat and remained as a distinct mass on the rim and ORL. Voluma was found in the deep medial cheek compartment and crossed into the superficial medial cheek and superficial nasolabial compartments and subcutaneous fat. In contrast, Restylane-LYFT remained mostly within the deep medial cheek compartment without spreading into the superficial fat pads (Figure). Dilutional manipulation by blending with saline and physical manipulation by massaging both increased spread of all products within the anatomical planes. However, Restylane products appeared to remain distinctly within the anatomical planes they were injected in despite the manipulations.

**Conclusion:**

This cadaveric study was successful in demonstrating the behavior of different hyaluronic acid fillers injected in the infraorbital region. Tear trough and malar region filler injections just above the periosteum resulted in precise anatomical placement along the infraorbital rim/ORL and in the deep medial cheek fat compartment, respectively. The Juvéderm products appeared to spread across anatomical planes more freely than Restylane products. This may be explained by the lower elastic modulus (G’) of the Juvéderm fillers. Manipulation of the filler product including blending and post-injection massage appeared to spread the fillers medially and laterally within the injected anatomical planes compared to unmanipulated filler. Of note, there are limitations to this study including the cadaver model as this does not allow for hydrophilic swelling of the filler nor spread and integration of the products as in live patients. The results of this study provide information about filler products commonly used in the infraorbital region and the effects of manipulation using blending or massage. Confirmation of precise anatomical placement of the fillers and improved knowledge of their behavior in situ through this cadaver study may assist clinical practitioners’ selection of rheological properties of fillers, method of constitution, and manipulation technique depending on patient-specific anatomy.

